# A case of primary duodenal Brunner's gland hamartoma that gradually underwent morphological changes over a period of 10 years

**DOI:** 10.1002/deo2.70028

**Published:** 2024-10-29

**Authors:** Yusuke Sunada, Hiromichi Yamane, Nobuaki Ochi, Hirohito Kirishi, Takako Saitou, Masafumi Miura, Hidekazu Nakanishi, Hideyo Fujiwara, Nagio Takigawa

**Affiliations:** ^1^ Department of General Internal Medicine 4 Kawasaki Medical School Okayama Japan; ^2^ Department of Pathology Kawasaki Medical School Okayama Japan

**Keywords:** Brunner's gland hamartoma, monitoring over 10 years, morphological change, natural course, prostate cancer

## Abstract

Brunner's gland hamartoma (BGH) is a benign tumor occurring in the duodenal bulb. BGH is typically asymptomatic, but it has been shown to occasionally cause anemia.

The patient was a 76‐year‐old male. In October 2011, he was diagnosed with prostate cancer with multiple bone metastases and was referred to us for the treatment and examination of anemia. Hormonal therapy with androgen receptor antagonists and bisphosphonate administration following orchiectomy improved his symptoms. In August 2012, esophagogastroduodenoscopy (EGD) was performed due to stomach discomfort, revealing a 5 mm semi‐pedunculated polyp in the duodenal bulb, Yamada‐Fukutomi classification type II. Over the next 5 years, the prostate cancer treatment proceeded smoothly, and no endoscopic follow‐up was conducted. In January 2017, during a health checkup, EGD revealed that the polyp in the duodenum bulb had changed morphologically with a distinct stalk measuring 10 mm. As there were no symptoms and only minimal tumor growth, a watchful waiting approach was adopted. In April 2022, due to the rapid progression of anemia, EGD was performed again, showing that the pedunculated polyp had enlarged to 20 mm in maximum diameter with an eroded surface and a stalk extending to 40 mm. Given the tumor enlargement and further examination of anemia, an endoscopic polypectomy was performed in May 2022. Histopathological examination confirmed the diagnosis of BGH. We observed a case of primary duodenal BGH during treatment for advanced prostate cancer, with endoscopic monitoring over 10 years. The morphological changes of BGH were clearly documented via EGD.

## INTRODUCTION

Brunner's gland hamartoma (BGH) is a benign tumor that typically arises in the duodenal bulb, most frequently occurring in middle‐aged adults in their 50s–60s. Previous reports indicate that BGH can cause anemia and gastrointestinal obstruction, and BGH is often discovered during investigations for these conditions. However, asymptomatic incidental findings of BGH are not uncommon.^1^ Here, we report a case of BGH identified during an anemia workup in a patient undergoing treatment for prostate cancer. The natural course of the BGH can be monitored endoscopically over a 10‐year period in this case.

## CASE REPORT

A 60‐year‐old male was diagnosed with prostate cancer with multiple bone metastases in October 2011. He was referred to us for further examination and treatment of anemia, and medical treatment of multiple bone metastases. The anemia was suspected to be mainly caused by hematopoietic dysfunction due to multiple bone metastases of prostate cancer. Following the orchiectomy, the patient received hormonal therapy with androgen receptor antagonists and bisphosphonates, which led to an improvement in anemia.

**FIGURE 1:**
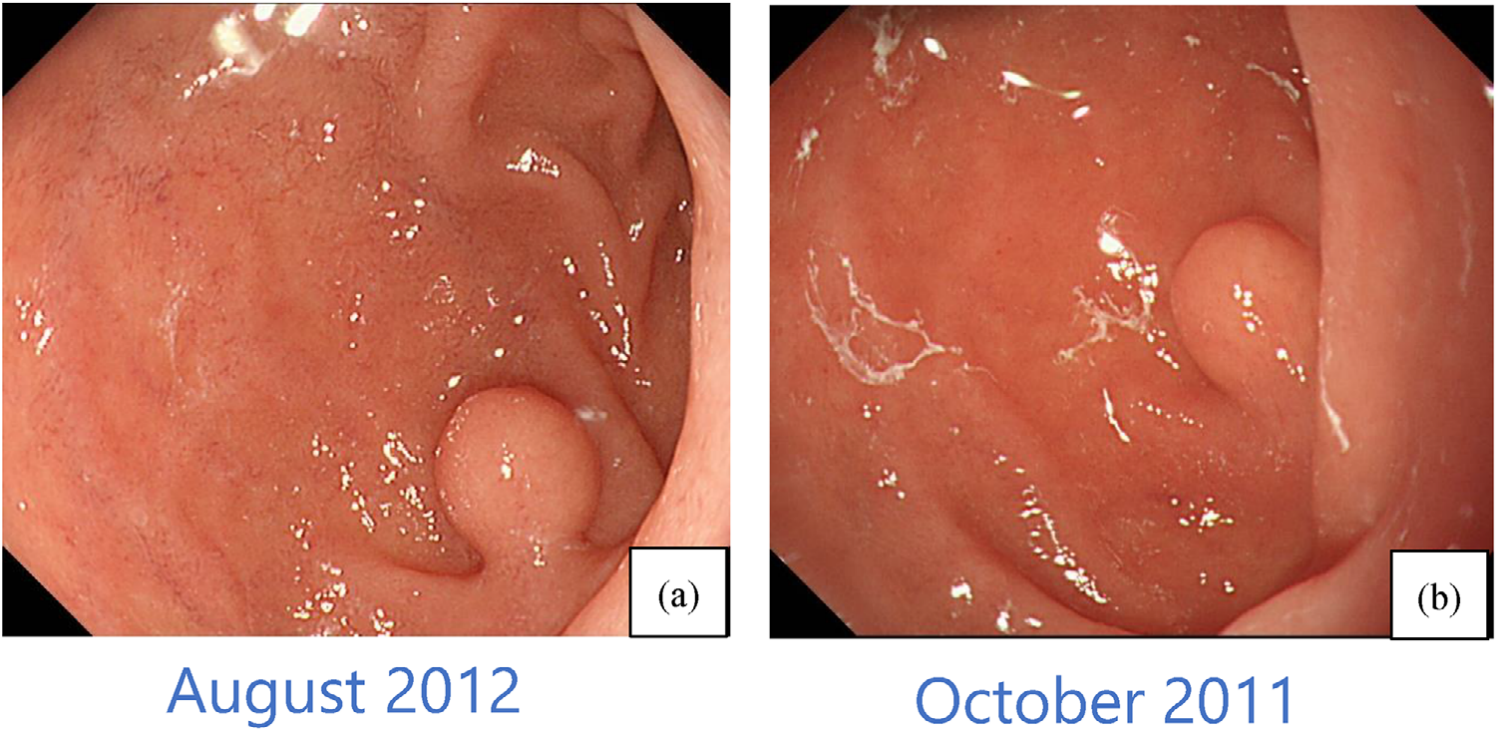
(a–b) In August 2012, esophagogastroduodenoscopy revealed that a type II semi‐pedunculated polyp of 5 mm in maximum diameter according to Yamada‐Fukutomi's classification was found in the duodenal bulb. Although there was no description of lesions in the same area during the esophagogastroduodenoscopy in October 2011, a confirmed polyp of nearly the same size was observed retrospectively.

In August 2012, due to stomach discomfort, an esophagogastroduodenoscopy (EGD) was performed. A type II semi‐pedunculated polyp of 5 mm in maximum diameter according to the Yamada‐Fukutomi's classification was found in the duodenal bulb (Figure 1a,b). Although a submucosal tumor was considered a possibility based on the endoscopic findings, a biopsy revealed only inflammatory changes, ruling out a duodenal metastasis of prostate cancer. The treatment for prostate cancer proceeded smoothly for the following 5 years, and therefore, follow‐up with EGD for the duodenal polyp was not conducted.

**FIGURE 2:**
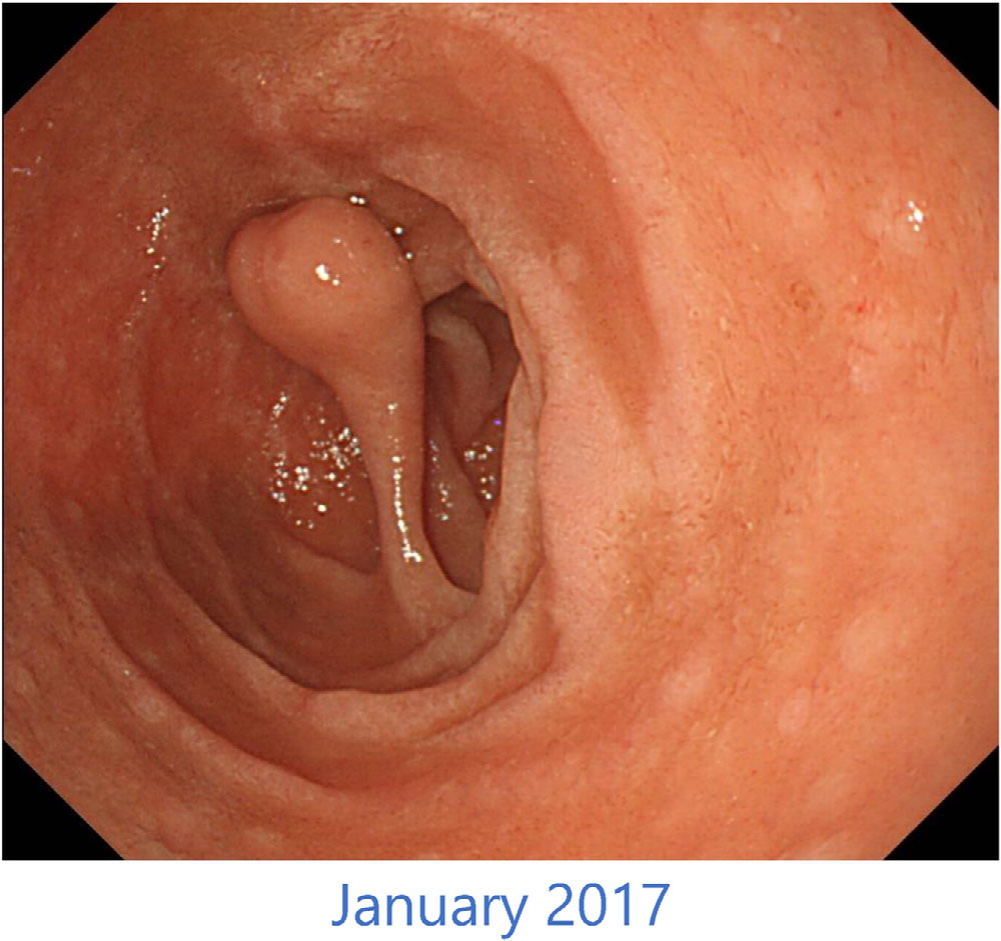
In January 2017, esophagogastroduodenoscopy revealed that the duodenal polyp had morphologically changed to a type 4 polyp according to Yamada‐Fukutomi's classification. The maximum diameter of the polyp was 8 mm, and it had transformed into a pedunculated polyp with a distinct stalk measuring 15 mm.

In January 2017, when an EGD was performed as part of a health check‐up, the duodenal polyp had morphologically changed to a type 4 polyp according to Yamada‐Fukutomi's classification. The maximum diameter of the polyp was 8 mm, and it had transformed into a pedunculated polyp with a distinct stalk measuring 15 mm (Figure 2). At that time, there were no clinical symptoms, and over the 5 years, only minimal tumor growth was observed. Consequently, no additional treatment was administered, and regular monitoring continued. Subsequently, prostate cancer gradually developed resistance to hormone therapy. Chemotherapy, including estramustine phosphate and docetaxel, was administered to manage prostate cancer.

**FIGURE 3:**
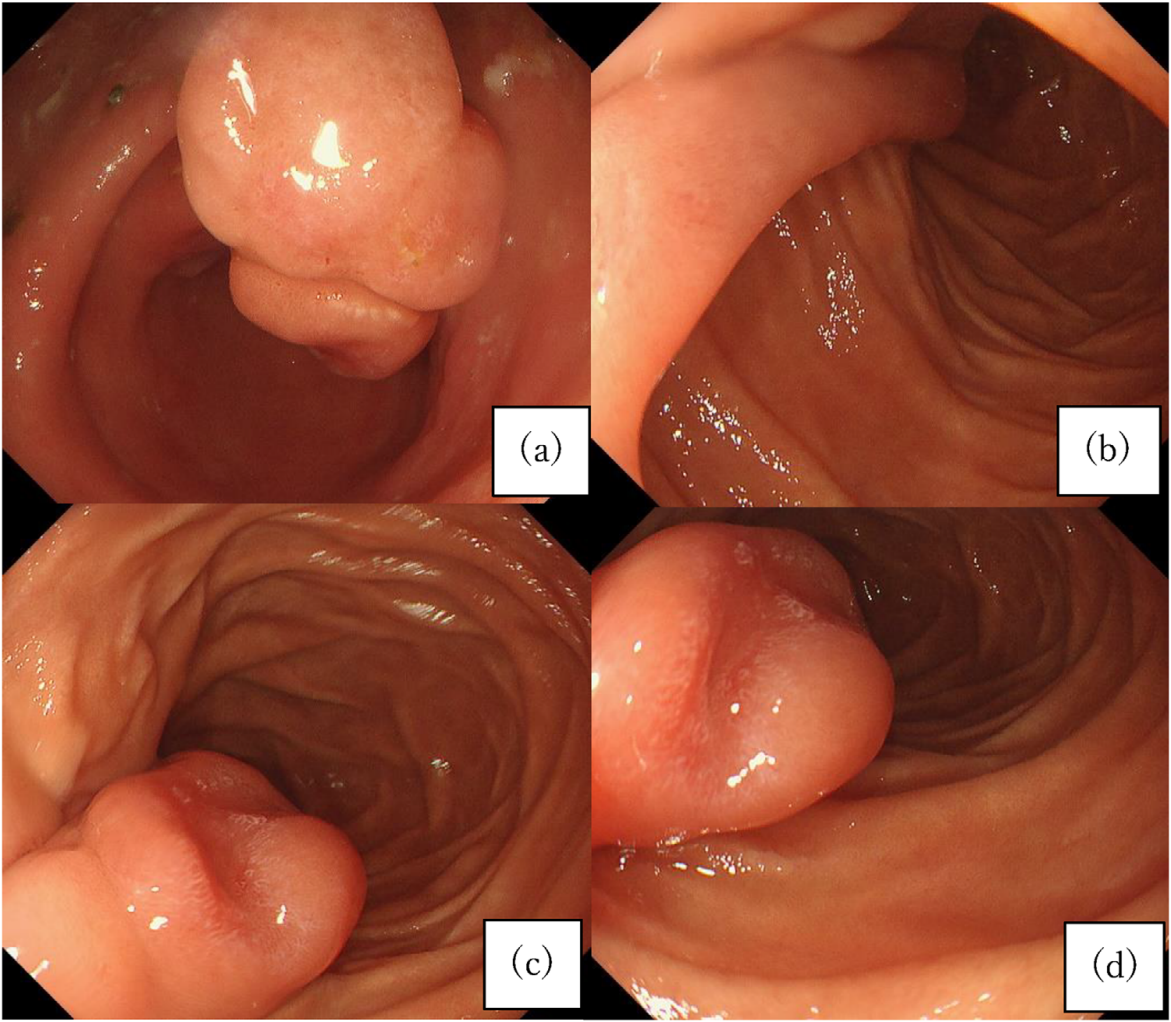
(a–d) The duodenal polyp had increased to a maximum diameter of 20 mm, with erosion on the apex, and the stalk had elongated to 40 mm.

In April 2022, the patient experienced a sudden progression of anemia, prompting an EGD to investigate the source of bleeding. The duodenal polyp had increased to a maximum diameter of 20 mm, with erosion on the apex, and the stalk had elongated to 40 mm (Figure 3a–d). Given the gradual tumor growth and morphological changes over 10 years and the possibility that the duodenal polyp was the source of bleeding (Supplementary Figure 1), it was also necessary to rule out metastatic lesions of prostate cancer and primary malignant tumors of the duodenum. During the endoscopic examination, we also considered differential diagnoses including Brunner's gland hyperplasia (BGH), an unusual form of metastatic prostate cancer, lymphoid follicular hyperplasia, and duodenal adenoma. However, the pathological diagnosis using a biopsy specimen indicated normal tissue with inflammation.

**FIGURE 4:**
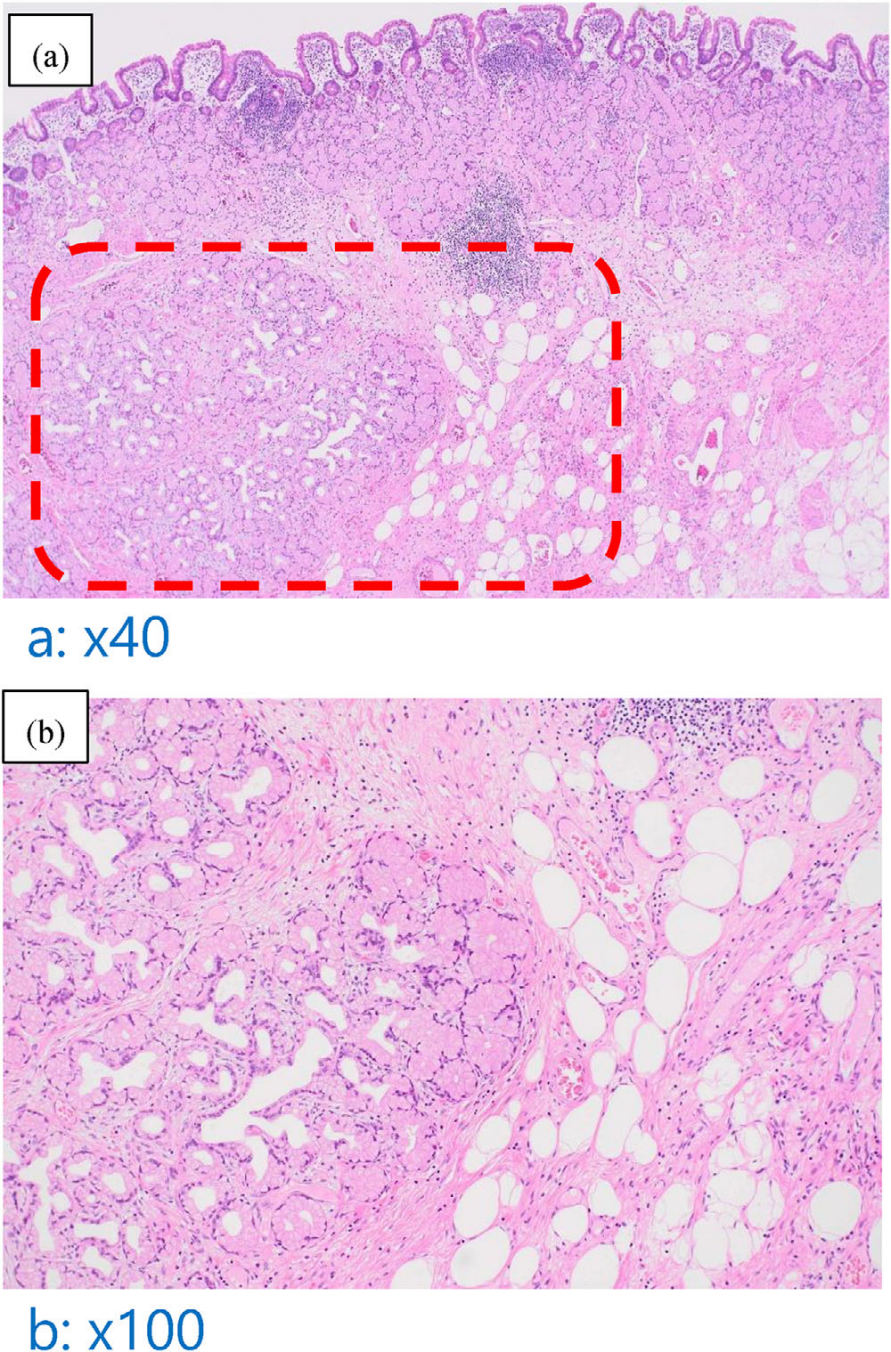
(a–b) Pathological findings: The histological examination revealed that the mass exhibited lobulated hyperplasia of Brunner's glands, partitioned by smooth muscle bundles, with interspersed adipose tissue and lymphocytic aggregates.

We performed polypectomy for both diagnostic and therapeutic purposes in May 2022. The base of the stalk was located in the duodenal bulb, and the stalk itself measured 40 mm in size. First, the tumor was pulled into the stomach using forceps, followed by a thorough observation of the tumor lesion. Subsequently, a detachable snare was secured at the base of the stalk and tightened. Then, a high‐frequency snare was applied slightly above the detachable snare, ligated, and the stalk was excised (Figure S2). Pathological examination of the resected specimen diagnosed the duodenal polyp as Brunner's gland hyperplasia (Figure 4a and b). Since anemia improved after the removal of the polyp, it is possible that the enlargement of BGH, though not definitive, may have been one of the causes of anemia. The course of the anemia is shown in Figure S1.

## DISCUSSION

Since its initial report in 1876, BGH has been frequently documented as a primary benign tumor of the duodenal bulb. It generally accounts for about 5% of all duodenal tumors and commonly occurs in middle‐aged and elderly individuals, specifically in their 50s and 60s, with a favorable prognosis. ^2^ The primary causes of its discovery are upper gastrointestinal bleeding and gastrointestinal obstruction, though incidental findings during medical checkup are not uncommon. The tumor size of BGH and Brunner's gland hyperplasia has been reported to range broadly from 5 to 120 mm.^1–4^ BGH is often covered by normal duodenal mucosa pathologically, making diagnosis through conventional endoscopic biopsy challenging. Therefore, many cases require definitive diagnosis through endoscopic polypectomy or surgical specimens. ^4^ In our case, a definitive diagnosis was not achieved with the endoscopic biopsy performed in 2012, but was confirmed through endoscopic polypectomy conducted in 2022. Since the maximum diameter was around 20 mm, endoscopic treatment was chosen, though larger tumors often necessitate surgical resection due to the difficulty of endoscopic manipulation within the duodenal bulb.^5.6^ This case was diagnosed as BGH following repeated investigations of anemia that included EGD. Numerous studies have reported the investigation of anemia as a common reason for BGH discovery^1^, ^2^, ^3^, ^4^, ^5^, ^6^ (Table S1).

However, in our case, the patient had advanced prostate cancer with evident multiple bone metastases as a background factor. The degree of anemia was often influenced by prostate cancer treatment, necessitating multiple endoscopic examinations for anemia. This allowed for the early detection of the BGH at a size of 5 mm before it could grow to a size capable of causing gastrointestinal bleeding. Additionally, the slow progression of prostate cancer, despite its advanced stage, due to appropriate treatment, enabled a long‐term observation of the natural course of BGH over 10 years. Over the span of 10 years, significant changes were observed in the endoscopic findings. BGH might take a considerable amount of time to manifest clinical symptoms as part of its natural course. To date, numerous reports and discussions have been made regarding factors such as the reasons for discovery and treatment options for BGH. However, our presented case suggests that in addition to these factors, consideration of the time axis may also be necessary. In our presented case, it took more than 10 years for the BGH to enlarge from 5 to 20 mm, and if there had been no progression of anemia, endoscopic resection might not apply. The value of this case seems to lie in the suggestion that observation, rather than resection, could have been a viable option. To the best of our knowledge, the natural course of BGH over such a long period has never been reported.

The pathological diagnosis of BGH is challenging, particularly in distinguishing it from Brunner's gland hyperplasia. BGH contains smooth muscle, Brunner's glands, and fat components, and they typically lack a well‐defined capsule.^1,5,6^ The key point in distinguishing between BGH and Brunner's gland hyperplasia is whether elements other than Brunner's glands, such as smooth muscle and fat components, are present within the lesion. In this case, smooth muscle and adipose tissue are observed in Figure 4, suggesting a diagnosis of BGH.

In conclusion, we encountered a case of BGH in a duodenal bulb observed over a 10‐year natural course while undergoing EGD during treatment for advanced prostate cancer. The morphological changes of the BGH were clearly revealed by EGD. This case is considered to be significant for understanding the natural course of BGH.

## CONFLICT OF INTERESTS STATEMENT

None.

## ETHICS STATEMENT

‐ Approval of the research protocol by an Institutional Reviewer Board (N/A)

‐ Informed Consent – The written consent of the patient has been obtained from the patient.

‐ Registry and the Registration No. of the study/trial (N/A)

‐ Animal Studies (N/A)

## Supporting information




**FIGURE S1** Due to the progression of anemia, an upper gastrointestinal endoscopy (esophagogastroduodenoscopy [EGD]) was performed, followed by a polypectomy. Subsequently, the anemia gradually showed signs of improvement.

**FIGURE S2** A detachable snare was secured at the base of the stalk and tightened. Then, a high‐frequency snare was applied slightly above the detachable snare, ligated and the stalk was excised.

**TABLE S1** Summary of reports about Brunner's gland hamartoma.
