# Supplementary figures and images for: A case of primary duodenal Brunner's gland hamartoma that gradually underwent morphological changes over a period of 10 years
Source: DEN Open. 2024 Oct 29;5(1):e70028. doi: 10.1002/deo2.70028 (PMC11522027; doi:10.1002/deo2.70028)

**Supplementary Figure 1**

**
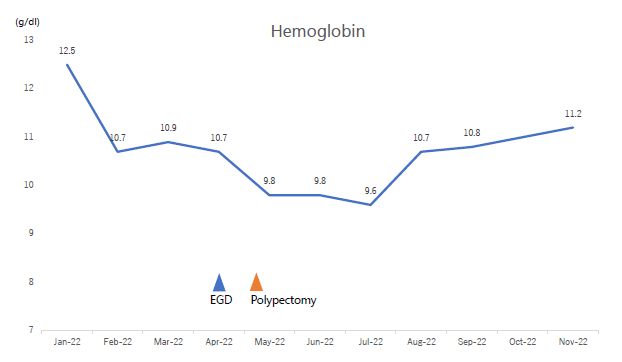
**

Supplement: Supplementary file 1 — FIGURE S1 Due to the progression of anemia, an upper gastrointestinal endoscopy (esophagogastroduodenoscopy [EGD]) was performed, followed by a polypectomy. Subsequently, the anemia gradually showed signs of improvement. [file DEO2-5-e70028-s001.docx]

**Supplementary Figure 2**


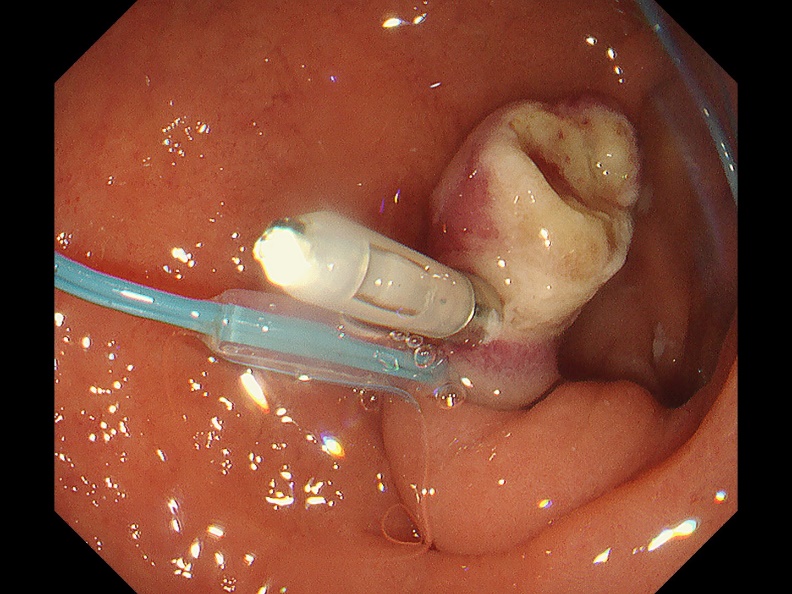

Supplement: Supplementary file 2 — FIGURE S2 A detachable snare was secured at the base of the stalk and tightened. Then, a high‐frequency snare was applied slightly above the detachable snare, ligated and the stalk was excised. [file DEO2-5-e70028-s002.docx]
