# Supplementary material for: A case of primary duodenal Brunner's gland hamartoma that gradually underwent morphological changes over a period of 10 years
Source: DEN Open. 2024 Oct 29;5(1):e70028. doi: 10.1002/deo2.70028 (PMC11522027; doi:10.1002/deo2.70028)
Supplement: Supplementary file 3 — TABLE S1 Summary of reports about Brunner's gland hamartoma. [file DEO2-5-e70028-s003.docx]

Supplementary Table

| **Author & Patient’s number** | **Reference** | **Types of Manuscript** | **Symptoms** | **Size** | **Treatment** | **Location** |
| --- | --- | --- | --- | --- | --- | --- |
| Levine JA et al  N=27 | Ref 1 | Review | Bleeding: 10  Obstruction: 10  Incidental: 7 | 10 mm to 60 mm | Surgical resection: 24  Endoscopic resection: 3 | First portion: 27 |
| Lin JC et al  N=1 | Ref 2 | Case report | Bleeding | 50 mm | Surgical resection | Second portion |
| Krishnamurthy P et al  N=12 | Ref 3 | Review | Obstruction: 12  Bleeding: 2  (Multiple answers  can be provided) | 20 mm to 120 mm | Not clarified | First portion: 6  Second/Third: 5  Unknown: 1 |
| Lee JH et al  N=1 | Ref 5 | Case report | Bleeding | 93 mm | Endoscopic resection | First portion |
| Stolpman DR et al  N=1 | Ref 6 | Case report | Bleeding | 45 mm | Surgical resection | Second portion |

Table　Summery of reports about Brunner’s gland hamartoma
